# Novel exotic alleles of *EARLY FLOWERING 3* determine plant development in barley

**DOI:** 10.1093/jxb/erad127

**Published:** 2023-04-03

**Authors:** Tanja Zahn, Zihao Zhu, Niklas Ritoff, Jonathan Krapf, Astrid Junker, Thomas Altmann, Thomas Schmutzer, Christian Tüting, Panagiotis L Kastritis, Steve Babben, Marcel Quint, Klaus Pillen, Andreas Maurer

**Affiliations:** Institute of Agricultural and Nutritional Sciences, Chair of Plant Breeding, Martin Luther University Halle-Wittenberg, Betty-Heimann-Str. 3, D-06120 Halle (Saale), Germany; Institute of Agricultural and Nutritional Sciences, Martin Luther University Halle-Wittenberg, Betty-Heimann-Str. 5, D-06120 Halle (Saale), Germany; Institute of Agricultural and Nutritional Sciences, Chair of Plant Breeding, Martin Luther University Halle-Wittenberg, Betty-Heimann-Str. 3, D-06120 Halle (Saale), Germany; Institute of Agricultural and Nutritional Sciences, Chair of Plant Breeding, Martin Luther University Halle-Wittenberg, Betty-Heimann-Str. 3, D-06120 Halle (Saale), Germany; Department of Molecular Genetics, Leibniz Institute of Plant Genetics and Crop Plant Research (IPK) Gatersleben, Stadt Seeland, Germany; Department of Molecular Genetics, Leibniz Institute of Plant Genetics and Crop Plant Research (IPK) Gatersleben, Stadt Seeland, Germany; Institute of Agricultural and Nutritional Sciences, Chair of Plant Breeding, Martin Luther University Halle-Wittenberg, Betty-Heimann-Str. 3, D-06120 Halle (Saale), Germany; Interdisciplinary Research Center HALOmem, Charles Tanford Protein Center, Martin Luther University Halle-Wittenberg, Kurt-Mothes-Str. 3a, D-06120 Halle (Saale), Germany; Interdisciplinary Research Center HALOmem, Charles Tanford Protein Center, Martin Luther University Halle-Wittenberg, Kurt-Mothes-Str. 3a, D-06120 Halle (Saale), Germany; Institute of Biochemistry and Biotechnology, Martin Luther University Halle-Wittenberg, Kurt-Mothes-Str. 3, D-06120 Halle (Saale), Germany; Biozentrum, Martin Luther University Halle-Wittenberg, Weinbergweg 22, D-06120 Halle (Saale), Germany; Institute of Agricultural and Nutritional Sciences, Martin Luther University Halle-Wittenberg, Betty-Heimann-Str. 5, D-06120 Halle (Saale), Germany; Institute of Agricultural and Nutritional Sciences, Martin Luther University Halle-Wittenberg, Betty-Heimann-Str. 5, D-06120 Halle (Saale), Germany; German Centre for Integrative Biodiversity Research (iDiv), Halle-Jena-Leipzig, Germany; Institute of Agricultural and Nutritional Sciences, Chair of Plant Breeding, Martin Luther University Halle-Wittenberg, Betty-Heimann-Str. 3, D-06120 Halle (Saale), Germany; Institute of Agricultural and Nutritional Sciences, Chair of Plant Breeding, Martin Luther University Halle-Wittenberg, Betty-Heimann-Str. 3, D-06120 Halle (Saale), Germany; CSIRO Agriculture and Food, Australia

**Keywords:** Alphafold2, disorder-to-order transition, *EARLY FLOWERING 3* (*ELF3*), flowering time, HEB-25, heterogeneous inbred family (HIF), *Hordeum vulgare*, phase separation, plant development, wild barley

## Abstract

*EARLY FLOWERING 3* (*ELF3*) is an important regulator of various physiological and developmental processes and hence may serve to improve plant adaptation which will be essential for future plant breeding. To expand the limited knowledge on barley *ELF3* in determining agronomic traits, we conducted field studies with heterogeneous inbred families (HIFs) derived from selected lines of the wild barley nested association mapping population HEB-25. During two growing seasons, phenotypes of nearly isogenic HIF sister lines, segregating for exotic and cultivated alleles at the *ELF3* locus, were compared for 10 developmental and yield-related traits. We determine novel exotic *ELF3* alleles and show that HIF lines, carrying the exotic *ELF3* allele, accelerated plant development compared with the cultivated *ELF3* allele, depending on the genetic background. Remarkably, the most extreme effects on phenology could be attributed to one exotic *ELF3* allele differing from the cultivated Barke *ELF3* allele in only one single nucleotide polymorphism (SNP). This SNP causes an amino acid substitution (W669G), which as predicted has an impact on the protein structure of ELF3. Consequently, it may affect phase separation behaviour and nano-compartment formation of ELF3 and, potentially, also its local cellular interactions causing significant trait differences between HIF sister lines.

## Teaser

Novel naturally occurring exotic alleles of *EARLY FLOWERING 3*(*ELF3*) are determined and associated with agronomic traits, suggesting a central role for *ELF3*in barley adaptation.

## Introduction

The performance of crops such as barley depends on their ability to adapt to different environments, which ultimately determines the yield potential. In the context of an ever growing world population and climate change, maximizing crop yields for further food supply will be pivotal (FAOSTAT, 2009) and could be ensured, for example, by adaptation of crops to different environments (Challinor *et al.*, 2014). More precisely, a meta-analysis of crop yield under climate change and adaptation based on 1700 studies even predicted that cultivar adaptation would be the most promising way to increase yield under the predicted climate change (Challinor *et al.*, 2014). Plant features important for plant adaptation are tolerance or resistance to abiotic and biotic stress factors such as water and nutrient availability, extreme temperatures, and soil salinity, as well as pathogen infections. To maximize grain yield by adaptation, the exact timing of plant development and flowering time are particularly important (Cockram *et al.*, 2007; Francia *et al.*, 2011; Alqudah and Schnurbusch, 2017; Wiegmann *et al.*, 2019; Fernandez-Calleja *et al.*, 2021), for instance by adjusting the phenological stages to avoid periods of extreme stress (Zheng *et al.*, 2013; Kazan and Lyons, 2016; Cammarano *et al.*, 2021).

Flowering time is mainly controlled by day length and vernalization (Turner *et al.*, 2005; Andres and Coupland, 2012). To adjust flowering time, plants therefore need to be able to react to changes in photoperiod and temperature. For adaptation of barley cultivation to a wider range of environments, early flowering genotypes are necessary for short growing seasons, while late flowering increases yield in temperate climates (Cockram *et al.*, 2007; Fernandez-Calleja *et al.*, 2021). The response to photoperiod under long-day conditions in barley is mainly controlled by *PPD-H1*, a pseudo-response regulator, which promotes *VRN-H3*, a homologue of *Arabidopsis thaliana* (Arabidopsis) *FLOWERING LOCUS T* (*FT*), through *CONSTANS* (*CO*), but also independently of *CO*, leading to the induction of flowering (Turner *et al.*, 2005; Yan *et al.*, 2006; Campoli *et al.*, 2012a; Faure *et al.*, 2012). Vernalization requirement is mainly controlled by the interaction of *VRN-H1* (Yan *et al.*, 2003), and *VRN-H2* (Yan *et al.*, 2004), both affecting *VRN-H3*. While *VRN-H2* represses *VRN-H3*, *VRN-H1* is up-regulated during vernalization, leading to the activation of *VRN-H3* and repression of *VRN-H2*, which in turn leads to the interruption of *VRN-H2*-regulated *VRN-H3* repression, promoting the induction of flowering (Yan *et al.*, 2006; Hemming *et al.*, 2008; Deng *et al.*, 2015). Due to a natural deletion of the entire *VRN-H2* gene, spring barley lacks the vernalization requirement (Yan *et al.*, 2004; von Zitzewitz *et al.*, 2005; Hemming *et al.*, 2008). Furthermore, there are genotypes that do not respond to photoperiod or vernalization, making it possible to expand crop cultivation even further north. These genotypes have been characterized with *early maturity* (*eam*) or *earliness* per se (*eps*) loci (Faure *et al.*, 2012). These loci may bring a new source of variation for the adaptation to different environments (Campoli and von Korff, 2014).

Flowering time is a complex trait which is controlled by a large regulatory network (Blümel *et al.*, 2015). A central role in this network is taken by *EARLY FLOWERING 3* (*ELF3*), which is the focus of this study. ELF3 is an integral part of the circadian clock in both Arabidopsis and barley (Zagotta *et al.*, 1996; Faure *et al.*, 2012; Zakhrabekova *et al.*, 2012; Müller *et al.*, 2020). In general, the circadian clock is necessary to react and adapt to daily and seasonal environmental changes (Wijnen and Young, 2006; Harmer, 2009). It regulates a number of important genes that control plant growth processes and thereby contributes significantly to plant performance of important agronomic traits and adaptation to different environments (Nusinow *et al.*, 2011; Bendix *et al.*, 2015; Calixto *et al.*, 2015).

The mechanistic understanding of the circadian clock is mainly based on studies in the model plant Arabidopsis, where *ELF3* functions as a core component of the clock (Thines and Harmon, 2010; Nusinow *et al.*, 2011). Arabidopsis *ELF3* (*AtELF3*) is an oscillating gene with an expression peak in the early evening. *AtELF3* encodes a multifunctional protein that in turn regulates various physiological and developmental processes (Hicks *et al.*, 2001; Nusinow *et al.*, 2011), for example by repressing the activity of further core circadian clock genes (Dixon *et al.*, 2011). Due to its diverse protein–protein interaction networking capabilities, AtELF3 presumably functions as a hub (Huang *et al.*, 2016). Together with ELF4 and LUX ARRYTHMO (LUX), AtELF3 forms the evening complex (EC), a transcriptional regulator, which is an integral part of the circadian clock, repressing clock- and growth-associated transcription factors (Nusinow *et al.*, 2011; Huang and Nusinow, 2016). For loss-of-function *AtELF3* mutants, an early flowering phenotype was shown (Zagotta *et al.*, 1996) and, in the context of this analysis, it is important to note that AtELF3 controls photoperiod-responsive growth and flowering time, as well as temperature responsiveness of the circadian clock (Anwer *et al.*, 2020; Jung *et al.*, 2020; Zhu *et al.*, 2022).

In barley (*Hordeum vulgare*), several clock orthologues from Arabidopsis have been identified with a high degree of conservation (Campoli *et al.*, 2012b; Calixto *et al.*, 2015; Müller *et al.*, 2020). The gene *Praematurum-a* (*Mat-a*)/*EARLY MATURITY 8* (*EAM8*) was identified as a barley homologue of *AtELF3* (Faure *et al.*, 2012; Zakhrabekova *et al.*, 2012), from then on denoted as *HvELF3*. Its influence on flowering seems to be conserved since barley plants with a loss-of-function *elf3* also show early flowering phenotypes. Furthermore, those plants are insensitive to photoperiod and their circadian rhythm is disrupted (Faure *et al.*, 2012; Zakhrabekova *et al.*, 2012; Boden *et al.*, 2014). Also, HvELF3 has recently been identified as a core component of the circadian oscillator since its absence leads to a non-rhythmic expression of other clock components (Müller *et al.*, 2020), making it an essential regulator of the clock also in barley. Faure *et al.* (2012) have shown that *elf3* mutations lead to a higher expression of *PPD-H1*, particularly during the night, which subsequently induces *VRN-H3* and thereby earlier flowering. Also, under long-day conditions, variation at *PPD-H1* was shown to influence flowering time of *elf3* mutants (Faure *et al.*, 2012). Furthermore, in *elf3* mutants, altered expression of core clock and clock-output genes (*CO*, *VRN-H3*, *CIRCADIAN CLOCK ASSOCIATED1*, *GIGANTEA*, and *TIMING OF CAB EXPRESSION1*) has been observed, and increased expression of *HvFT1* (*VRN-H3*) was observed independently of *PPD-H1* (Faure *et al.*, 2012; Boden *et al.*, 2014). Ejaz and von Korff (2017) have shown later flowering under high ambient temperature for the cultivar Bowman, which harbours a functional *HvELF3* allele, whereas, for an introgression line with a non-functional *HvELF3* allele in a Bowman background, flowering time was accelerated. Furthermore, a larger reduction in floret and seed number has been observed for Bowman under high ambient temperature than for the introgression line. As such, *ELF3* (or natural variants/mutants thereof) contributed significantly to barley domestication and adaptation to higher latitudes by conferring a day-neutral flowering phenotype.

**Fig. 1.**
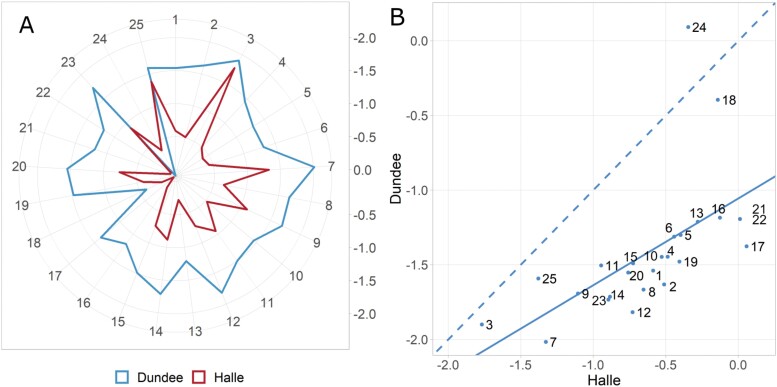
Family-specific effect diversity of exotic *ELF3* (*ELF3*_*Hsp*_) alleles compared with the cultivated *ELF3* (*ELF3*_*Hv*_) alleles for the trait heading in days. Comparison of all 25 families of the barley nested association mapping (NAM) population HEB-25 from Herzig *et al.* (2018). (A) Each line of the radar plot shows the respective HEB family with its average *ELF3*_*Hsp*_ effect on flowering time, compared with the *ELF3*_*Hv*_ allele, in days [radar from inside to outside: no acceleration (0) to an acceleration of 2 d (–2)]. (B) Scatterplot comparing *ELF3*_*Hsp*_ flowering time effects in days between the two different locations Halle and Dundee. The regression line is shown as a solid line and the dashed line is the diagonal separating effect strengths between the two locations. Pearson’s correlation coefficient between locations is 0.6.

All barley research mentioned above is based on *elf3* loss-of-function mutants. We wanted to explore the role of natural barley *ELF3* variants, which is why we used the nested association mapping (NAM) population ‘Halle Exotic Barley’ (HEB-25). The population originates from crosses of 25 highly divergent wild barley accessions (*Hordeum vulgare* ssp. *spontaneum* and *agriocrithon*, hereafter abbreviated as *Hsp*) with the elite cultivar Barke (*Hordeum vulgare* ssp. *vulgare*, hereafter abbreviated as *Hv*). A previous study on plant development traits in barley identified a quantitative trait locus (QTL) region containing the *ELF3* locus (Maurer *et al.*, 2016). This QTL significantly affected the traits shooting (SHO; Zadok’s stage Z31), shoot elongation phase (SEL; time from Z31 to Z49), heading (HEA; Z49), maturity (MAT; Z87), and plant height (HEI). Herzig *et al.* (2018) also describe the potential effects of *ELF3* on the traits SHO, HEA, MAT, and HEI, confirming the results from our previous study in a different environmental context. In both studies, the exotic *ELF3* alleles (*ELF3*_*Hsp*_) accelerated plant development and decreased plant height compared with the cultivated *ELF3* allele (*ELF3*_*Hv*_). Furthermore, *ELF3*_*Hsp*_ effects for the mentioned traits varied for the 25 HEB families. Results for QTL1H10 (128–133.1 cM), the respective QTL for *ELF3*, were extracted from Herzig *et al.* (2018) and are shown in Fig. 1 for the trait HEA. Here, the exotic *ELF3*_*Hsp*_ has varying effects among HEB families, but it is, in most cases, accelerating flowering (up to 2 d) compared with the cultivated barley *ELF3*_*Hv*_ allele. Except for family 24, *ELF3*_*Hsp*_ effects were stronger in Dundee (UK). Here, the maritime climate in 2014 and 2015 was characterized with colder summers, more and equally distributed rain, and greater day lengths compared with Halle (Germany) with moderate-to-continental growing conditions (Herzig *et al.*, 2018). The contrasting effects between the two field locations suggested that the *ELF3*_*Hsp*_ effect on heading depends on environmental cues. Furthermore, Herzig *et al.* (2019) mentioned *ELF3*_*Hsp*_ as potentially affecting grain nutrient content.

However, despite these associations, there are few causal data about the effect of natural *ELF3* variants on barley flowering time regulation and crop performance. Xia *et al.* (2017) already described 51 *ELF3* haplotypes in 134 accessions (several cultivars, landraces, and wild barleys, mainly native to the Qinghai–Tibet Plateau). Of these, the novel *eam8.l* mutation was assumed to be responsible for an early flowering phenotype because of a mutation at position 3257 which probably contributed to intron retention and a truncated protein. However, other haplotypes also exhibited early flowering phenotypes, indicating the existence of other reasons for early flowering (Xia *et al.*, 2017). As the selection of independent mutations at the *ELF3* locus might be a valuable tool to adapt barley cultivation to a wider range of environments (Faure *et al.*, 2012), the aim of this study was to investigate the influence of further natural barley *ELF3* variants on several developmental and yield-related traits to subsequently identify *ELF3* alleles, which, in turn, may lead to an improvement of barley performance across environments. For this purpose, the barley NAM population HEB-25 was used as a basis for selection of heterogeneous inbred families (HIFs). HIFs can be derived from advanced generations of lines with initial heterozygosity at a genomic region of interest. In this manner, allele effects can be estimated in a nearly isogenic background (Tuinstra *et al.*, 1997; Bergelson and Roux, 2010). HEB-25 offers a diverse panel of wild barley alleles in a cultivated Barke background (Maurer *et al.*, 2015). HIFs can be derived from those expected 6.25% of BC_1_S_3_ lines being heterozygous at *ELF3* to examine its association with a phenotype, enabling a direct comparison of allele effects on traits between two nearly isogenic HIF sister lines segregating for the homozygous exotic and cultivated genotypes at *ELF3*.

Besides time to flowering (HEA), additional phenological traits were investigated such as time to shooting (SHO), duration of shoot elongation (SEL), duration of ripening phase (RIP; time from Z49 to Z87), and time to maturity (MAT). Furthermore, plant height (HEI), ears per square metre (EAR), grain number per ear (GNE), thousand grain weight (TGW), and grain yield (YLD) were investigated. Here, we describe significant effects of exotic *ELF3* variants on several agronomic performance traits, making *ELF3* an attractive target for future climate-resilient breeding approaches in barley. By investigating the *ELF3* coding sequences, we determine novel exotic *ELF3* alleles and show that a single nucleotide polymorphism (SNP) in one exotic *ELF3* allele promotes expression of *VRN-H1*, potentially through a predicted altered protein structure of ELF3, which might reshape the phase separation behaviour and nano-compartment formation of ELF3.

## Materials and methods

### Plant material

For this study, HIFs were selected from the multiparental barley NAM population HEB-25 (Halle Exotic Barley; Maurer *et al.*, 2015), which consists of 1420 individual BC_1_S_3_ lines that were developed by an initial cross of the spring barley cultivar Barke (*H. vulgare* ssp. *vulgare*) with 25 highly divergent wild barley accessions (*H. vulgare* ssp. *spontaneum* and *agriocrithon*). For detailed information about the population design, see Maurer *et al.* (2015). In this study, HIF pairs were derived from HEB lines in generation BC_1_S_3:11_, which were heterozygous at *ELF3* in generation BC_1_S_3_. In addition, HIF pair 25_002_BC2 originates from a backcross of HEB line 25_002 (BC_1_S_3:7_), carrying the *ELF3*_*Hsp*_ allele, with Barke. Here, the HIF pair was selected from the segregating progeny of the resulting BC_2_ plant. With the chosen plants (11 HIF pairs from nine HEB families), two field trials were conducted in 2019 and 2020.

### Genotyping of HEB lines and HIF pairs

For a pre-selection of potential HIFs, existing Infinium iSelect 50k SNP genotype data of HEB-25 were used (Bayer *et al.*, 2017; Maurer and Pillen, 2019). Physical positions of SNPs were derived from the Morex reference sequence v2 (refseq2) (Monat *et al.*, 2019). SNP data were first checked for quality, then an identity-by-state (IBS) matrix was created, coding homozygous Barke alleles as 0 and homozygous wild alleles as 2. Accordingly, heterozygous lines were coded as 1. Subsequently, the IBS matrix was converted to an identity-by-descent (IBD) matrix, as described in Maurer *et al.* (2017). This resulted in 32 995 SNP markers, which were used for pre-selection. Hereby, the first selection criterion was heterozygosity at the locus of interest, the *ELF3* gene (HORVU.MOREX.r2.1HG0078390.1). A gene-specific marker (JHI_Hv50k_2016_57670), which is located inside *ELF3*, and flanking markers were used to determine whether heterozygosity was present at *ELF3* (Supplementary Table S1). Furthermore, lines showing heterozygosity at one of the other seven major flowering time loci in barley (Maurer *et al.*, 2015) were discarded from the pre-selection, to ensure that no additional segregation in the background of *ELF3* would compromise the effect estimation of *ELF3* on traits, especially flowering time. Fifty plants of each BC_1_S_3:11_ line were grown and genotyped with kompetitive allele-specific PCR (KASP) markers covering the *ELF3* region (Supplementary Table S2) (Semagn *et al.*, 2014) at TraitGenetics, Gatersleben, to select an *ELF3* HIF pair made of two nearly isogenic lines segregating for *ELF3*_*Hv*_ and *ELF3*_*Hsp*_. During the field trial in 2019, the genotypes of selected HIF pairs were validated by TraitGenetics with the barley Infinium iSelect 50k chip (Bayer *et al.*, 2017) and subsequently converted to an IBD matrix as described above (Supplementary Table S3).

### Field trials

In both years, 2019 and 2020, field trials were conducted at the ‘Kühnfeld Experimental Field Station’ of Martin Luther University Halle-Wittenberg (51°29ʹ46.47″N, 11°59ʹ41.81″E) to gather phenotypic data for 11 selected HIF pairs. Both field trials were sown in March (4 March 2019 and 19 March 2020), with fertilization and pest management carried out according to local practice. In 2019, the field trial was conducted in a randomized complete block design consisting of four blocks, each containing a randomized replication of the 11 selected HIF pairs. The plots consisted of three rows (50 seeds each) with a length of 1.5 m and a distance of 0.15 m between rows. Plots were evenly spaced by 0.3 m. The field trial in 2020 was conducted in six randomized blocks. The plots consisted of eight rows with a length of 3.2 m, a distance of 0.15 m between rows, 0.3 m between plots, and a seeding density of 300 seeds m^–2^. Both sister lines of a HIF pair were always sown next to each other for comparison and to minimize spatial effects. Additionally, elite donor Barke was placed as a control in 27 plots in 2019 and 11 plots in 2020.

### Environments

The growth period had the same length in both years, except that in 2020 sowing was carried out 2 weeks later than in 2019 (4 March 2019 and 19 March 2020) as earlier sowing was not possible due to too much rain and wet soil in 2020. Therefore, maturity of the latest line was 2 weeks earlier in 2019. During the respective growth periods of the field trials, the mean temperature was 0.5 °C higher in 2020 (13.4 °C); in the third month of the vegetation period, in particular, when heading started, the temperature was on average 3 °C higher in 2020. However, during the last month of the respective growth period, the temperature was almost 2 °C higher in 2019 (21.3 °C) with high daily average temperatures of up to 29 °C. In 2020, the highest daily average temperature was 23.8 °C (Supplementary Fig. S1; Supplementary Table S4). The sum of precipitation over the whole growth period was almost the same in both years (~127 mm). While in 2019 rainfalls occurred during spring, directly after sowing and equally distributed over the summer, almost no rain occurred during the first third of the vegetation period and almost 50% of rain during the last month of the vegetation period in 2020 (Supplementary Fig. S1; Supplementary Table S4). Due to later sowing in 2020, the day was 1 h longer at the beginning of the experiment in 2020 and the longest day (21 June) was later in the vegetation period in 2019 than in 2020, leading to a larger absolute amount of daylight in 2020 [cumulative day length (CDL): 1775:42 h in 2020 compared with 1715:42 h in 2019, Supplementary Table S4].

### Phenotypic data

Phenotypic data were recorded in both years for 10 developmental and yield-related traits (Table 1). For the developmental traits SHO until MAT, growing degree days (GDD) were calculated following equation (1) of McMaster and Wilhelm (1997) with a base temperature of 0 °C. The decision as to which trait days or GDD was used is based on estimated repeatabilities (Rep) and heritabilities (H^2^) (Supplementary Protocol S1; Supplementary Table S5).

**Table 1.** List of evaluated traits

Abbreviation	Trait	Units	Method of measurement
SHO	Time to shooting	Days	Number of days from sowing until first node noticeable 1 cm above tillering node (Z31; Zadoks *et al.*, 1974) for 50% of all plants of a plot.
SEL	Shoot elongation phase	GDD	Time from SHO to HEA.
HEA	Time to heading	Days	Number of days from sowing until first awns are visible (Z49; Zadoks *et al.*, 1974) for 50% of ears on main tillers of a plot.
RIP	Ripening phase	Days	Time from HEA to MAT.
MAT	Time to maturity	GDD	Number of days from sowing until hard dough: grain content firm and fingernail impression held (Z87; Zadoks *et al.*, 1974) for 50% of all plants of a plot.
HEI	Plant height	cm	Average plant height of all plants of a plot at maturity; measured from ground to tip of erected ear (without awns).
EAR	Ears per m^2^		Number of ears m^–2^; counted by using a representative 50 cm frame in the centre of a plot and extrapolated to 1 m^2^.
GNE	Grain number per ear		Number of grains per ear; based on a representative sample of 10 harvested ears and recorded with the MARVIN seed analyser (GTA Sensorik, Neubrandenburg, Germany).
TGW	Thousand grain weight	g	Weight of 1000 grains; extrapolated after harvest with MARVIN seed analyser based on a representative sample of 10 ears. Before, seeds were cleaned and damaged seeds were sorted out.
YLD	Grain yield	dt ha^–1^	For each plot, total grain yield was calculated based on the yield parameters EAR, GNE, and TGW and extrapolated to dt ha^–1^.

### Image-based phenotyping in controlled environments

To validate barley *ELF3* effects in a controlled environment, a phenotyping experiment was conducted using the LemnaTec system at the Leibniz Institute of Plant Genetics and Crop Plant Research (IPK) in Gatersleben. One HIF pair (10_190), the cultivar Bowman, and two *elf3* mutants in a Bowman background [BW289 and BW290, carrying the *eam8.k* and *eam8.w* alleles, respectively (Faure *et al.*, 2012; Zakhrabekova *et al.*, 2012)], were grown in 13–15 replications per genotype for 64 d (sowing on 18 November 2019) under standard conditions with day/night temperatures of 20 °C/18 °C and long days (LDs) with 16 h light and 8 h darkness, respectively. Top- and side-view RGB images (LemnaTec automatic phenotyping system at IPK Gatersleben) from each plant were taken every day after day 8 and every 2–4 d after day 33. All analysed growth and developmental parameters were scored from these images. To obtain data for plant height, area, and volume, the Integrated Analysis Platform (IAP) pipeline was used (Klukas *et al.*, 2014). The number of tillers was counted manually on day 64 (all tillers were included). On day 64, the aerial parts of the plants were harvested, and fresh weight was measured using an electronic scale. Dry weight was measured after placing plant material into a drying oven for 3 d at 80 °C.

### Statistical analyses

All statistical analyses were performed with either SAS 9.4 (SAS Institute Inc., Cary, NC, USA) or R 3.4.3 (R Development Core Team, Vienna, Austria). Basic descriptive statistics and comparative statistics between HIF sister lines were calculated in R using the compare_means method ANOVA. SAS PROC HPMIXED was used to estimate best linear unbiased estimators (BLUEs), assuming fixed genotype and block effects in a linear mixed model. Pearson’s correlation coefficients were calculated using the CORRGRAM package in R. BLUEs were used for calculation of correlation of traits across years, and individual values were used for correlation of traits within a year.

Repeatabilities (Rep) for each year and broad-sense heritabilities (H^2^) were calculated as


$$\begin{array}{l} Rep=\frac{V_{G}}{V_{G}+\frac{V_{F}}{r}}and\;\;\;H^{2}=\frac{V_{G}}{V_{G}+\frac{V_{GY}}{y}+\frac{V_{F}}{yr}}, \end{array}$$

where V_G_, V_GY_, and V_F_ correspond to the genotype, genotype×year, and error variance components, respectively. The terms y and r represent the number of years and replicates, respectively. For estimation of variance components with SAS procedure PROC VARCOMP, all effects were assumed to be random. Furthermore, an ANOVA was conducted for each trait to test for significant genotype and year effects as well as for significant genotype×year interactions. For the image-based trial, ANOVA was conducted pairwise with SAS to test for significant trait effects between Bowman and the two mutants as well as between the two HIF sister lines of 10_190 for plant height, area, and volume. For the traits heading, number of tillers, fresh weight, and dry weight, ANOVA was conducted to test for significant differences between all five genotypes (pairwise).

### ELF3 coding and protein sequence

The full-length genomic sequence of *ELF3*, from original wild barley donors, Barke, Bowman, and BW290, was amplified using Ex Taq DNA Polymerase (Takara Bio, Kusatsu, Shiga, Japan). The purified amplicons were submitted to Eurofins Genomics (Ebersberg, Germany) for dideoxy sequencing. Five to six overlapping fragments were assembled, and the coding sequence was then obtained by alignment with the reported *ELF3* sequence from cultivar Igri (GenBank accession number HQ850272; Faure *et al.*, 2012). Subsequently, the protein sequences were obtained by using the ExPASy translate tool (Gasteiger *et al.*, 2003). In addition, the promotor region of *ELF3* in HIF sister lines of 10_190 was sequenced. Therefore, genomic DNA was amplified using ALLin™ RPH Polymerase from highQu (https://www.highqu.com/ALLin-RPH-Polymerase/HLE0101) and purified via the Thermo Scientific™ GeneJET PCR Purification Kit (https://www.thermofisher.com/order/catalog/product/K0701) before it was Sanger sequenced by Eurofins Genomics. Primers used for PCR and sequencing are given in Supplementary Table S6.

The structure of Barke *HvELF3* was visualized by using Exon–Intron Graphic Maker available at http://wormweb.org/exonintron. AtELF3 protein (Col-0) was obtained at https://www.arabidopsis.org/ [AT2G25930, The Arabidopsis Information Resource (TAIR)] and three AtELF3 protein domains were defined according to Nieto *et al.* (2015). For alignment with AtELF3, Barke ELF3 was obtained as described above and the Morex ELF3 sequence from Morex reference sequence v2 (Monat *et al.*, 2019). Alignment of AtELF3 and HvELF3 (of Barke/Morex) sequences was done using MAFFT version 7 (Kuraku *et al.*, 2013; Katoh *et al.*, 2019) available at https://mafft.cbrc.jp/alignment/server/, and subsequently the respective HvELF3 protein domains were retrieved.

Furthermore, for comparison purposes of the coding sequence variation in the HEB-25 wild donors with already described variation in the literature, *ELF3* coding sequences were extracted from Xia *et al.* (2017) as well as from the NCBI (https://www.ncbi.nlm.nih.gov/) and Jayakodi *et al.* (2020), using the IPK Galaxy Blast suite (https://galaxy-web.ipk-gatersleben.de/). Determination of *ELF3* haplotypes based on exon sequences was done using the ‘haplotypes’ package in R.

### Gene expression analysis

To test for differences in gene expression between HIF sister lines, HIF pairs 10_003 and 10_190 as well as plants of Bowman and BW290 were grown for 16 d under LDs with 16 h light and 8 h darkness, day/night temperatures of 20 °C/18 °C, and a light intensity of 300 μmol m^–2^ s^–1^. On day 17, starting from the onset of light (ZT00), leaf samples were harvested every 4 h, using three biological replicates. Total RNA was isolated using the NucleoSpin RNA Plant Kit (Macherey-Nagel), cDNA was synthesized using the PrimeScript RT Reagent Kit (Perfect Real Time, Takara Bio), and quantitative real-time PCRs were performed on an AriaMx Real-Time PCR System (Agilent) using Absolute Blue Low Rox Mix (Thermo Fisher Scientific). The reference genes *HvGAPDH* and *PTF* were used for normalization. Primer sequences are described in Supplementary Table S6. Differences between Bowman and BW290, or between HIF sister lines at each time point were analysed for significance by a two-sided Student’s *t*-test using GraphPad QuickCalcs (http://graphpad.com/quickcalcs/).

### ELF3 protein sequence structure analysis

For global structure prediction, a local installation of Alphafold v2.1.0 (Jumper *et al.*, 2021) with max_template_date=2021-10-12 and model_preset=monomer_ptm was used. Results were analysed by an in-house python script (available upon request).

To identify structural homologues, the BLASTp webserver with the database ‘Protein Data Bank proteins (pdb)’ and the ELF3 subsequences ‘SSRGSELQWSSAASSPFDRQ’ and ‘SSRGSELQGSSAASSPFDRQ’ were used. The derived hits were analysed by an in-house PyMOL script (available upon request) regarding their structural completeness (minimum of five resolved residues in the pdb file) and their annotated secondary structure. The weblogo was generated using the Berkley weblogo webserver (Crooks *et al.*, 2004).

For disorder analysis, the amino acid sequences of the barley homologues from the annotated Arabidopsis proteins were identified using BLASTp. A local installation of the MobiDB-lite suite (Necci *et al.*, 2017) was used to predict the disorder content of the derived sequences.

## Results and discussion

### Phenotypic variation suggests year-by-year analysis

We observed broad variation for all traits, between both genotypes and years (Supplementary Fig. S2; Supplementary Tables S7, S8), with medium high coefficients of variation (CVs) in both years. As expected, for the elite parent and control cultivar Barke, the CV was not as high as the CV across the studied HIF pairs. CVs for YLD were particularly high, which can be explained by the high variation of EAR. An ANOVA revealed significant (*P*<0.001) effects for genotype and year as well as for genotype×year interaction, except for SEL, MAT, and TGW between the two years and RIP, EAR, TGW, and YLD for genotype×year interaction (Supplementary Table S9). In 2020, for all developmental traits, except RIP and SEL [GDD], plants showed a faster development than in 2019 (Supplementary Table S8). For the trait SEL, plants showed a faster development in 2020 when comparing this growth phase in days, while GDD values were lower in 2019, showing that the average temperature in 2020 was higher during this growth period than in 2019. Furthermore, plants were smaller in 2020 and all yield components had lower values. In particular, yield was unexpectedly low in 2020. Presumably, the generally faster phenological development in 2020 led to a shorter growth period (e.g. due to different weather conditions, Supplementary Fig. S1; Supplementary Table S4) and left the plants less time for assimilation, grain filling, and biomass production, resulting in smaller plants, lower yield components, and, consequently, lower grain yield. The average grain yield for spring barley in Germany in 2020 was 55.6 dt ha^–1^ (Federal Ministry of Food and Agriculture, 2020). In this study, YLD was very high in 2019 (97.6 dt ha^–1^) whereas in 2020 it was far lower (38.8 dt ha^–1^). Due to the small plot size in 2019, yield was probably overestimated. Repeatabilities (Rep) for YLD confirm this fact, as Rep for YLD in 2019 is much lower than for YLD in 2020 (Supplementary Table S5). Barke, as a control, also confirms that, as it had a yield of 127.4 dt ha^–1^ in 2019 and 60.7 dt ha^–1^ in 2020. The latter amount is consistent with the average yield of 59.5 dt ha^–1^ for Barke in a previous study in Halle (Wiegmann *et al.*, 2019). In this case, HEB lines also showed lower yields than Barke.

Consequently, in addition to the ANOVA, the descriptive statistics emphasize the difference between the two trial years (Supplementary Fig. S2), meaning that differences in developmental and yield-related traits between the two years can mainly be explained by different environmental conditions (Supplementary Fig. S1; Supplementary Table S4; Supplementary Protocol S1). As a consequence, both years were evaluated separately. Correlations support this decision (Supplementary Figs S2, S3; Supplementary Protocol S1). High repeatabilities and heritabilities (Supplementary Table S5) indicate that the measurements were reliable (Supplementary Protocol S1). Furthermore, separate yearly evaluation is interesting since barley *ELF3* effects have already been shown to vary depending on the environment (Herzig *et al.*, 2018).

### Comparison of HIFs reveals effects between HIF sister lines

**Fig. 2.**
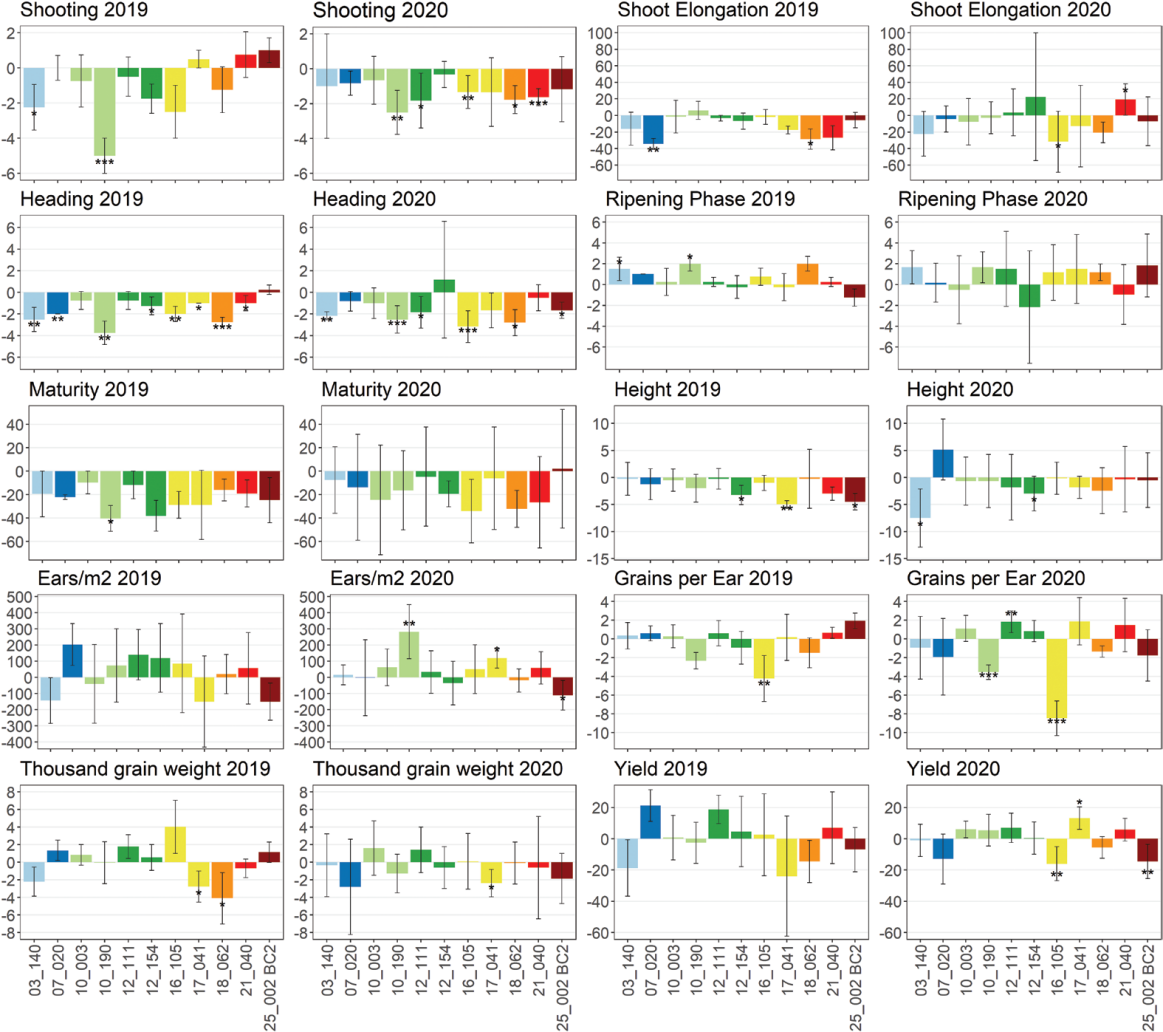
Trait differences between the two sister lines of each HIF pair (*ELF3*_*Hsp*_ compared with *ELF3*_*Hv*_) per year. Lines with two identical first digits originate from the same wild donor. Trait units are given in Table 1. Asterisks indicate a significant difference between sister lines (one-way ANOVA, *P<0.05, **P<0.01, and ***P<0.001) and error bars show SDs. Mean differences and SDs are based on differences for each HIF pair per block. Columns are coloured depending on the ELF3 haplotype defined in Fig. 4C.

Trait performance for each HIF line and the difference between HIF sister lines carrying the wild *ELF3*_*Hsp*_ and the elite *ELF3*_*Hv*_ alleles, respectively, were calculated per year (Supplementary Table S7; Fig. 2). Furthermore, descriptive statistics for each HIF sister line in each year can be found in Supplementary Table S10. For the sake of completeness, we also analysed data across years in Supplementary Table S7 and Supplementary Fig. S4.

In general, HIF sister lines carrying an *ELF3*_*Hsp*_ allele showed an accelerated plant development and reduced plant height in both years (Fig. 2). These findings confirm *Hsp* allele effects estimated by means of genome-wide association studies (GWAS) in previous trials (Maurer *et al.*, 2016; Herzig *et al.*, 2018). Also, family-specific effect variation of *ELF3*_*Hsp*_ alleles could be seen as in Herzig *et al.* (2018). Compared with Herzig *et al*. (2018), the effects observed in the current study are more diverse and more extreme. This is most likely to be due to the relatively rough effect estimation in the GWAS model of Herzig *et al.* (2018), especially as it is based on lines with different genetic backgrounds. In the current study, the effect estimates are thought to be more reliable, as the genetic background of HIF sister lines is almost identical. In contrast to the developmental traits, the yield parameters EAR, GNE, and TGW as well as YLD showed different effect directions between HIF sister lines.

Several significant effect differences were found, especially for SHO and HEA, most of which could be confirmed in both years. The strongest and greatest number of effects were found in HIF 10_190, where SHO was up to 5.00 d earlier for the *ELF3*_*Hsp*_ allele, for HEA up to 3.75 d earlier, and for MAT up to 40.39 GDD earlier, which corresponds to 1.75 d in 2019 (Supplementary Table S7). Significant effect differences in 10_190 could also be found for the traits RIP, EAR, and GNE. However, it should already be noted here, that although HIF 10_003 originates from the same wild donor (HID_102) as 10_190 and thus shares the same *ELF3*_*Hsp*_ allele, interestingly the strong phenotypic effects of 10_190 could not be observed in 10_003, indicating the presence of further factors determining the *ELF3*_*Hsp*_ allele effect differences and making it necessary to carry out an inspection of the genetic background (see ‘Genetic constitution of the HIF pairs beyond ELF3 impacts ELF3 effects’) and gene expression analysis (see ‘Gene expression analysis reveals that phenotypic differences are not due to ELF3 transcript abundance itself’).

Yield performance of all HIFs was different between the two years. In 2020, the absolute yield was far below average yields (Supplementary Table S7). Nevertheless, significant yield effects of the *ELF3*_*Hsp*_ allele-carrying lines were found for HIFs 16_105, 17_041, and 25_002 BC2, with yield differences of up to 15.96 dt ha^–1^ in HIF 16_105. In HIF 17_041, the *ELF3*_*Hsp*_-carrying line shows the strongest increase in yield (13.21 dt ha^–1^ in 2020). This is of great importance considering the absolute yield and the average yield for spring barley in Germany (55.6 dt ha^–1^ in 2020; Federal Ministry of Food and Agriculture, 2020). However, the correlation of earliness and yield largely depends on environmental conditions. A positive correlation of HEA and YLD in 2019 and a negative correlation in 2020 (Supplementary Fig. S3) confirm this and could be due to different temperature and/or precipitation resulting in a preference for later or earlier genotypes. Admittedly, a final evaluation of the influence of *ELF3* on yield would therefore require either optimal conditions without limiting factors or a series of field trials in a representative selection of environments.

Increasing yield has always been the main goal in plant breeding. Domestication and selection of crop plants improved yield but this was associated with loss of genetic diversity. Wild barleys provide a huge genetic resource that can be useful to extend the elite barley breeding pool to cope with challenges set by the ongoing climate change (Tanksley and McCouch, 1997; Ellis *et al.*, 2000; Zamir, 2001; Nevo, 2013). However, not only yield-improving genotypes are of interest for future breeding programmes, but also HIFs carrying exotic alleles for increasing biodiversity and improvement of other agronomic traits, provided that they are not associated with a yield penalty. This assumption also applies to plant height, since larger plants increase the risk of lodging and yield losses (Hedden, 2003). In this regard, depending on the environment, the *ELF3*_*Hsp*_-carrying HIF lines 10_190 and 12_111 may be useful for breeding. The exotic alleles exhibited significantly increasing effects on EAR or GNE (Fig. 2; Supplementary Fig. S4), respectively, without simultaneous negative effects on yield or plant height. Across years, 12_111, as the only line, even had a significantly positive effect on yield (Supplementary Fig. S4). If early heading is desired, the *ELF3*_*Hsp*_ alleles present in HIF lines 03_140 and 10_190 are interesting, since they showed early heading without negative effects on yield or plant height (Fig. 2; Supplementary Fig. S4).

However, to confirm that the effects found came from the respective *ELF3*_*Hsp*_ alleles, an analysis of the genetic background between HIF sister lines was conducted (see ‘Genetic constitution of the HIF pairs beyond ELF3 impacts ELF3 effects’).

### ELF3 effects in the context of environment

Generally, more significant trait effects were found in 2020 than in 2019 (Fig. 2). One reason could be that larger plots and extra replicates (six in 2020 versus four in 2019) are necessary to observe significant differences. Another reason could be that the *ELF3*_*Hsp*_ effect is larger under specific environmental conditions, as shown before (Herzig *et al.*, 2018). Herzig *et al.* (2018) reported that *ELF3*_*Hsp*_ effects on heading were stronger in Dundee (2014 and 2015) with colder summers (up to 16 °C on average), more and equally distributed rain (>800 mm), and greater day lengths (maximum of 17.45 h) compared with Halle. In Halle, the average temperature in July was up to 21 °C, 50% of the annual precipitation (514 mm) fell during July and August, and maximum day length was 16.63 h (Herzig *et al.*, 2018). In the present study, 2020 was characterized by a warmer vegetation period (on average 13.4 °C compared with 12.9 °C in 2019) except for the last month (on average 19.4 °C compared with 21.3 °C in 2019), with daily average temperatures of up to 23.8 °C (compared with up to 29 °C in 2019) and rain mainly at the end of the vegetation period instead of equally distributed rain as in 2019 (127 mm in both years during the vegetation period). Plants needed on average >11 d more to reach SHO and HEA in 2019 (Supplementary Table S8). Also, in 2020, days were longer than in 2019 for the major part of the growing season and the photoperiod (the absolute amount of daylight over the whole vegetation period, CDL) was higher compared with 2019 (Supplementary Table S4). In 2019, plants on average needed a >100 h longer photoperiod to reach SHO and HEA (Supplementary Table S8).

As a part of the circadian clock, controlling plant development based on day length and ambient temperature signals (Wijnen and Young, 2006; Harmer, 2009; Nusinow *et al.*, 2011; Bendix *et al.*, 2015; Calixto *et al.*, 2015), *ELF3* very probably plays a role in adaptation to environmental changes in barley. In Arabidopsis, the circadian clock is a major regulator of the response to abiotic stress (reviewed in Habte *et al.*, 2014). *ELF3*, as a part of the circadian clock, might also influence this in barley, as shown in Saade *et al.* (2016), where *ELF3*_*Hsp*_ effects were increased under salinity stress (for HEA, TGW, and HEI). *AtELF3* also controls growth in response to ambient temperature and photoperiod (Thines and Harmon, 2010; Box *et al.*, 2015; Raschke *et al.*, 2015; Anwer *et al.*, 2020; Jung *et al.*, 2020; Zhu *et al.*, 2022). It was suggested to support crop improvement under higher temperature (Zhu *et al.*, 2022). For barley, Ejaz and von Korff (2017) could show that a non-functional *elf3* leads to earlier flowering under high ambient temperature, whereas a functional *ELF3* leads to later flowering. Also, no reduction in floret and seed number was observed under high ambient temperature for a non-functional *elf3* compared with a functional *ELF3* allele.

Hence, we conclude that the environment in 2019 led to weaker effect differences, which could be caused by temperature, precipitation, and/or photoperiod effects (Supplementary Protocol S1). Therefore, a further experiment under controlled greenhouse conditions was conducted.

### Image-based phenotyping in a controlled environment validates results from field trials

To confirm the results from the field experiments in a different but typical experimental condition, HIF pair 10_190 was selected for a greenhouse experiment (LDs: 16 h light, 8 h darkness, day/night temperatures of 20 °C/18 °C) and compared with cultivar Bowman and the two *elf3* mutant lines BW289 and BW290. The latter were generated in a Bowman background, exhibiting early flowering phenotypes (Faure *et al.*, 2012; Zakhrabekova *et al.*, 2012; Ejaz and von Korff, 2017). HIF pair 10_190 was selected because it exhibited the strongest effects, especially for SHO and HEA, in both years in the field experiments (Fig. 2).

The method of phenotyping the traits heading, tiller number, and plant height was slightly different compared with the field trials. Here, heading was scored when the first awns of a plant appeared, which is well comparable with HEA in the field trials, where it was scored when the awns were visible for 50% of all plants of a plot. In the greenhouse, the number of tillers was counted manually on day 64 and all tillers were included, while in the field trials, the trait EAR was counted by using a representative 50 cm frame in the centre of a plot and only tillers actually carrying ears were counted. Plant height in the greenhouse was measured continuously and was obtained by analysing images and, in the field trial, it was solely measured at the end of maturity with tillers pulled upright.

**Fig. 3.**
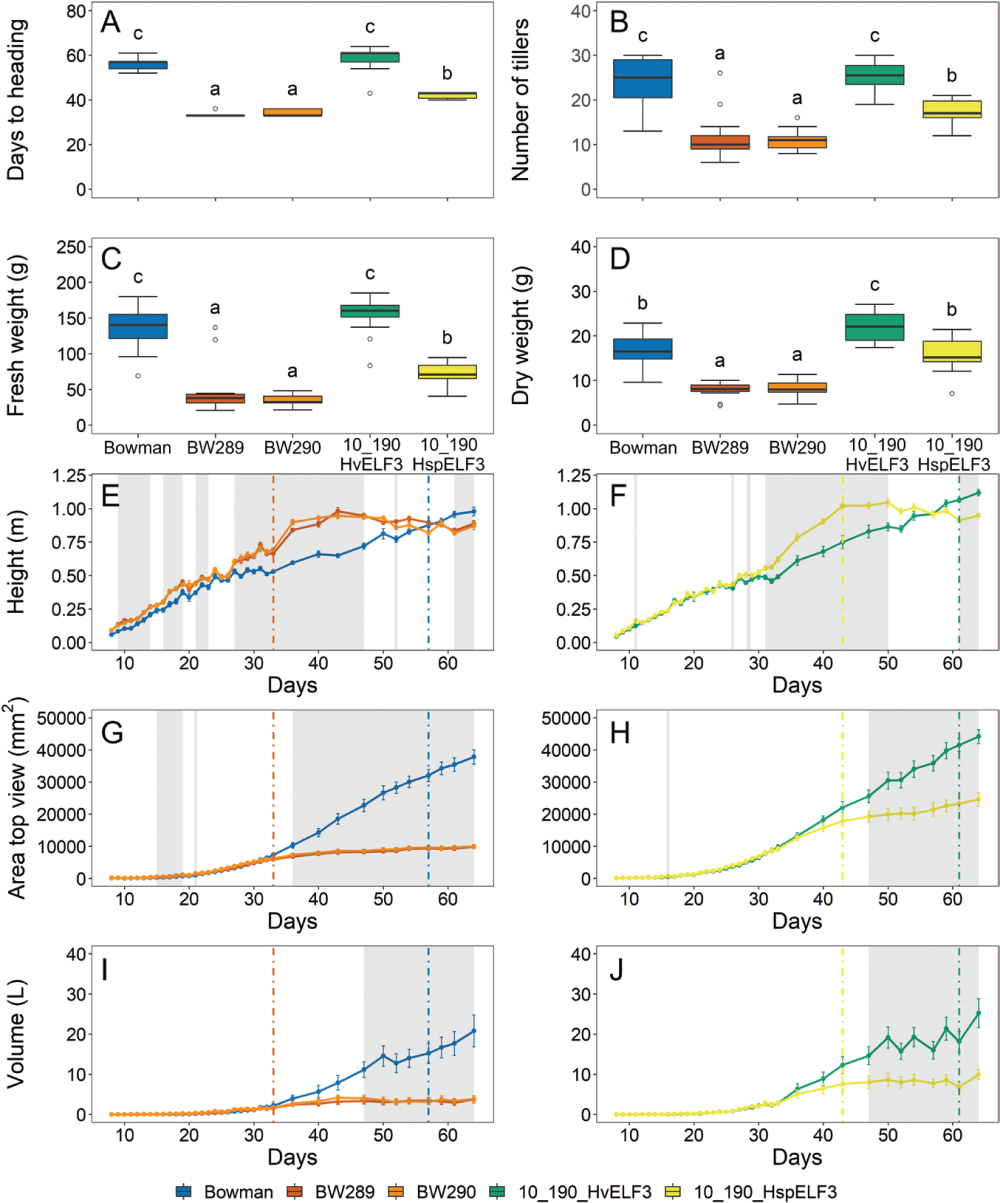
Growth and biomass parameters for cultivar Bowman, two *elf3* mutants in the Bowman background, BW289 and BW290, and HIF pair 10_190. Plants were grown under standard greenhouse conditions (LDs with 20 °C/18 °C day/night temperatures). Heading (A) was scored from images when the awn tips of the first awn were visible. Number of tillers (B), fresh weight (C), and dry weight (D) were measured at the end of the experiment (day 64). Boxplots (A–D) show medians and interquartile ranges (IQRs), and outliers were defined as 1.5× IQR. Different letters above boxes indicate significant differences (one-way ANOVA, *P*<0.05, Supplementary Table S11). Parameters height, area, and volume (E–J) were extracted from the Integrated Analysis Platform (IAP) pipeline (Klukas *et al.*, 2014). Coloured vertical lines show the mean flowering time of the respective genotype, and grey-shaded areas show significant differences for Bowman with both mutants (E, G, I) and between sisters lines of HIF 10_190 (F, H, J) (one-way ANOVA, *P*<0.05, Supplementary Table S11). Error bars indicate the SE of the mean across ≥13 biological replicates.

As expected, the mutants showed earlier flowering of ~24 d compared with Bowman (Fig. 3A). For the HIF pair, the line with the wild *ELF3*_*Hsp*_ allele flowered ~18 d earlier than the line carrying the *ELF3*_*Hv*_ allele, even outperforming the results of the field experiments and the previous QTL studies, probably due to the optimal conditions in the greenhouse (e.g. already 16 h of light at the beginning of the experiment and a constantly optimum temperature and water supply).

To evaluate whether barley *ELF3* had an impact in controlling vegetative growth, the three growth parameters plant height, area, and volume were measured or estimated (for volume) (Fig. 3E–J). Plant height showed an increase just before heading for both mutants and 10_190_*ELF3*_*Hsp*_, which could be related to the trait SHO from the field experiment where 10_190_*ELF3*_*Hsp*_ showed early shooting (Fig. 2). Just after heading, the growth curve flattened for the mutants (day 33) and 10_190_*ELF3*_*Hsp*_ (day 43), while growth of cultivar Bowman and 10_190_*ELF3*_*Hv*_ continued to increase. The same trend as for plant height was visible for plant area and plant volume (Fig. 3G–J) where the growth curve flattened for the mutants and 10_190_*ELF3*_*Hsp*_ directly after heading, whereas for Bowman and 10_190_*ELF3*_*Hv*_ growth strongly increased at the same time. These results confirm a reduced vegetative growth rate for BW289, BW290, and the wild barley 10_190_*ELF3*_*Hsp*_ allele. This is in accordance with the findings that BW289, BW290, and 10_190_*ELF3*_*Hsp*_ showed fewer tillers and lower fresh and dry weight compared with cultivar Bowman and 10_190_*ELF3*_*Hv*_ (Fig. 3B–D). This can also be explained by early heading and a shortened growth period (Fig. 3A). In the field experiment, no plant height effect was observed between sister lines of HIF 10_190 (Fig. 2). This may be explained by the fact that plant height in the field was measured at the end of maturity rather than during development. Strikingly, in the field experiment in 2020, the *ELF3*_*Hsp*_-carrying HIF 10_190 line had more ears per square metre compared with the *ELF3*_*Hv*_-carrying line. This effect could not be validated in the greenhouse experiment. A reason could be that for the greenhouse plants, all tillers were counted without considering if tillers would develop into a spike, whereas in the field experiments only developed ears were counted. In conclusion, the greenhouse results for 10_190 were able to confirm most of the results from the field trials, in particular for heading.

### High diversity in ELF3 protein sequences

**Fig. 4.**
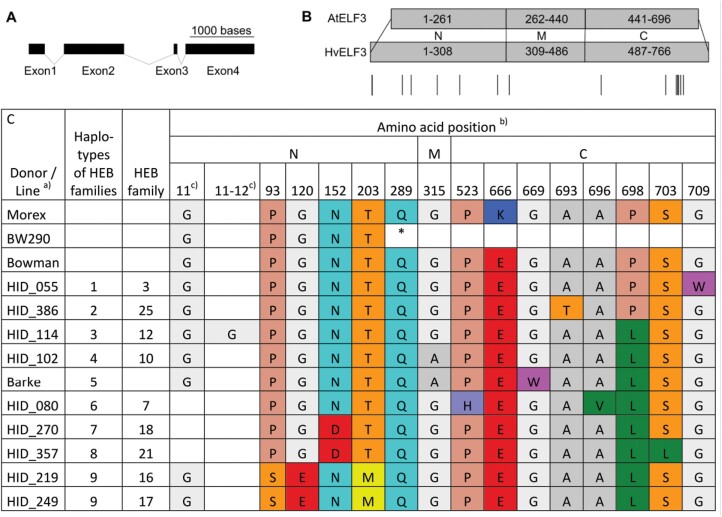
ELF3 protein structure and sequence polymorphisms. (A) Structure of the *HvELF3* gene in barley (Barke). Exons are shown as black rectangles and introns as connecting lines. (B) Domain mapping and their sequence annotation between the Arabidopsis (Col-0) ELF3 protein (AtELF3) and Barke/Morex ELF3 protein (HvELF3). Numbers indicate amino acid positions of N-terminal (N), middle (M), and C-terminal (C) protein domains. Amino acids 696 and 766 are the STOP codons for AtELF3 and HvELF3, respectively. Lines beneath HvELF3 mark sites of amino acid substitutions and insertion or deletion between HIFs used in field trials (as indicated in C). (C) ELF3 protein sequence polymorphisms of all alleles present in the field trials, Morex, Bowman, and BW290. Only the amino acid positions with variation between the families are shown. One-letter amino acid abbreviations (JCBN, 1984) were used, and the asterisk shows a stop codon. A) HID=‘hordeum identity’; name of the donor accession. b) N, M, and C regions of barley ELF3 were obtained by alignment of the Barke/Morex sequence with the Arabidopsis sequence, and Barke/Morex/Bowman sequences were used as references for the amino acid positions. c) Between position 11 and 12 some lines have an insertion and at position 11 some lines have a deletion of one amino acid, compared with the Barke amino acid sequence.

In order to understand the sequence variations of HvELF3 and to be able to better compare the Barke *ELF3*_*Hv*_- and wild *ELF3*_*Hsp*_ allele-carrying HIF sister lines with each other, we sequenced the full-length genomic DNA of *ELF3* (Fig. 4A) from original wild barley donors and Barke. After identifying the *ELF3* coding sequence of all wild barley donors (Supplementary Table S12), the ELF3 protein sequences were determined (Supplementary Table S13) and 19 different protein types/proteoforms could be distinguished (Supplementary Table S14), nine of which were present in the field trials (Fig. 4C) due to the above-mentioned selection criteria for HIF lines. Comparing the variation found in the wild donors of HEB-25 with already described variation for *HvELF3* in the literature revealed 23 novel mutations, of which 13 were novel non-synonymous SNPs (Supplementary Table S15). Furthermore, 19 *ELF3* alleles (out of 21 different *ELF3* haplotypes in donor lines of HEB-25) could be determined as novel alleles (Supplementary Table S15). N-terminal (N), middle (M), and C-terminal (C) regions of the HvELF3 protein were identified based on the comparison with AtELF3 (Fig. 4B), where these regions were shown to interact with different proteins (Liu *et al.*, 2001; Yu *et al.*, 2008; Herrero *et al.*, 2012; Nieto *et al.*, 2015).

In Arabidopsis, the N region is required to interact with PHYTOCHROME B (PHYB) and CONSTITUTIVE PHOTOMORPHOGENIC 1 (COP1) (Liu *et al.*, 2001; Yu *et al.*, 2008), the M region with ELF4 and GIGANTEA (GI) (Yu *et al.*, 2008; Herrero *et al.*, 2012), and the C region with PHYTOCHROME-INTERACTING FACTOR 4 (PIF4) (Nieto *et al.*, 2015). Huang *et al.* (2017) could already show that ELF3 in *Brachypodium distachyon*, a grass which is closely related to barley, interacts with almost the same set of proteins *in vivo*. While the mutation in BW289 (*eam8.k*) contains two deletions, one inversion, and two small insertions (Zakhrabekova *et al.*, 2012), BW290 (*eam8.w*) has a C-to-T point mutation, resulting in a premature stop codon (Fig. 4C), leading to truncated proteins in both mutants (Faure *et al.*, 2012). Since the M and C regions are absent in BW290 and since this line is flowering early (Faure *et al.*, 2012; Zakhrabekova *et al.*, 2012; Ejaz and von Korff, 2017), naturally occurring mutations in these regions may also influence the role of barley ELF3. Also, for the wild barley donors, most amino acid differences were observed in the N and C regions. Amino acid variation at positions 315, 669, and 698 were also described in Casas *et al.* (2021) for the two cultivars Beka and Logan and suggested to be associated with differences in flowering time. Apart from that, phenotypic differences are likely also to be sought on the *cis*-regulatory level.

A summary of all ELF3 protein polymorphisms present in the field trials and their respective phenotypic effects in both years (as in Fig. 2) can be found in Supplementary Table S16. Particularly interesting is that the donors of family 16 and 17 have exactly the same protein sequence, especially when considering the different effects on yield (Fig. 2), again indicating the presence of further factors in the remaining genome determining the *ELF3*_*Hsp*_ allele effects (see ‘Genetic constitution of the HIF pairs beyond ELF3 impacts ELF3 effects’; especially for families 16 and 17). Moreover, the exotic ELF3 in family 10 (HID_102) only differs in one amino acid from the cultivated ELF3 of Barke. This amino acid is located at position 669 in the C-terminal region of the ELF3 protein (Fig. 4C). In Arabidopsis, the C-terminal region of ELF3 binds the PIF4 basic helix–loop–helix (bHLH) domain which subsequently prevents PIF4 from activating its transcriptional targets (Nieto *et al.*, 2015). The *PIF4* gene in Arabidopsis controls thermomorphogenesis (Koini *et al.*, 2009; Quint *et al.*, 2016), which refers to morphological changes dependent on the ambient temperature. It regulates auxin biosynthesis, thermosensory growth, adaptations, and reproductive transition (Koini *et al.*, 2009; Franklin *et al.*, 2011; Kumar *et al.*, 2012; Gangappa *et al.*, 2017). Previous studies have shown that variation in *PIF4* expression and elongation growth can be explained by genetic variation in *AtELF3* (Box *et al.*, 2015; Raschke *et al.*, 2015). The mutation at position 669 has already been described in the literature for other accessions (Supplementary Table S15) and has been suggested to be associated with flowering time (Casas *et al.*, 2021). It should also be noted that Bowman showed the same phenotype as 10_190_*ELF3*_*Hv*_ in the greenhouse experiment (Fig. 3) although it shares the same W669G substitution as 10_190_*ELF3*_*Hsp*_ (Fig. 4C). Given that all other HIFs share this substitution (10_003 even harbours the exact same *ELF3*_*Hsp*_ without showing strong phenotypic effect differences) strengthens the assumption that there are further factors determining the *ELF3*_*Hsp*_ allele effect differences, such as structural variation on the protein level or in the remaining genome as already suggested previously. Therefore, we compared the genetic background of all HIFs and conducted gene expression analyses for *ELF3* and downstream genes in HIF 10_003 and 10_190, and performed a sequence-/structure-based analysis of the ELF3_Hv_ and ELF3_Hsp_ proteins from family 10 (see below).

### Genetic constitution of the HIF pairs beyond ELF3 impacts ELF3 effects

To find out whether the effects found between HIF sister lines were indeed due to *ELF3*_*Hsp*_ alleles, or may have been influenced by differences in the remaining genome, an inspection of the genetic background in HIF sister lines was carried out using the data from the 50k iSelect SNP chip (Supplementary Table S3). The genotyping results confirmed the status of the fixed homozygous *ELF3* alleles in all HIF sister lines. For the additional seven main flowering time loci found in the previous HEB-25 QTL studies (Maurer *et al.*, 2015), it was possible to verify that HIF sister lines exhibited the same fixed homozygous alleles (Supplementary Table S17).

Initially, we aimed for HIF pairs that would only segregate at the *ELF3* locus, but additional segregating loci between the HIF sister lines were obtained (Supplementary Fig. S5; Supplementary Table S18). Genes in these regions could possibly interfere with and have an influence on the studied traits and mask the *ELF3*_*Hsp*_ effect. In this context, the yield-related effects in HIF sister lines of 16_105 and 17_041, which seemed interesting due to the presence of the same *ELF3* allele, can now be explained by the presence of different brittle rachis (*btr1/btr2*) alleles (physical position of the region: chromosome 3H: base pairs 38 758 057–39 626 379 (refseq2), closest SNP: SCRI_RS_146425, Supplementary Table S3), which affect the shattering of the ear at maturity. Thus, the observed significant yield effects are due to a differing number of harvested grains per ear and have to be attributed to a brittle rachis phenotype (Pourkheirandish *et al.*, 2015) rather than to the *ELF3* difference. Apart from these two lines [16_105_959 (*ELF3*_*Hsp*_) and 17_041_1000 (*ELF3*_*Hv*_)], all other HIF lines carry cultivated alleles at *btr1*/*btr2* (Supplementary Table S3).

However, for a selection of genes that are already known to control flowering time or to interact with AtELF3, for example LUX and PIF4 (Nusinow *et al.*, 2011; Nieto *et al.*, 2015), most of the studied HIF pairs already share the same homozygous alleles (Supplementary Table S19). Nevertheless, there could be genes that are still not known to be involved in the flowering time control pathway and the circadian clock. Of course, as few as possible additionally segregating loci are desirable. In this context, HIF pairs derived from HEB-25 lines 10_003, 10_190, 12_111, and 21_040 with only a few additional segregating regions are especially interesting (<1% of the whole genome, Supplementary Table S18). Together with the results from the field trials (Fig. 2) and the greenhouse experiment (Fig. 3), the low percentage of segregation (Supplementary Table S18, S20) makes HIF 10_190 especially interesting.

HIFs 10_003 and 10_190 originate from the same exotic donor just like HIFs 12_111 and 12_154. Comparing these HIFs with each other regarding their genomic background revealed contrasting alleles at five further flowering time loci for HIFs from family 10 [*PPD-H1*, *CEN*, *QFt.HEB25-4a*, *VRN-H1*, and *VRN-H3/FT1* (Maurer *et al.*, 2015)] (Supplementary Table S17), and at two further flowering time loci for HIFs from family 12 [*PPD-H1* and *VRN-H1* (Maurer *et al.*, 2015)], (Supplementary Table S17). Also for other genes that are known to be involved in controlling flowering time, contrasting alleles were found for two *GI*-related genes, *LUX*, *ELF4*, and *PIF4* for HIFs in family 10 and for one *GI*-related gene, *LUX*, *CO2*, *ELF4*, *PPD-H2*, and *PIF4* in family 12 (Supplementary Table S19).

In the case of the HIFs in family 10, *PPD-H1* and *PIF4* are of special interest as W669G lies within the potential PIF4-interacting C region of ELF3 (Fig. 4C). In the case of *PPD-H1*, the wild allele has shown the strongest influence on flowering time and also on other traits (Maurer *et al.*, 2016; Herzig *et al.*, 2018). Here, the *ELF3*_*Hsp*_ effect might be increased in the presence of a homozygous wild *PPD-H1* allele, suggesting an interaction of these two. A previous study has already shown increased expression of *PPD-H1* in *elf3* mutants and effects on flowering time in *elf3* mutants by variation at *PPD-H1* under LDs (Faure *et al.*, 2012). To verify the differences in gene expression between HIF sister lines, we measured diurnal gene expression of *ELF3* and some of its downstream genes, including *PPD-H1*.

### Gene expression analysis reveals that phenotypic differences are not due to ELF3 transcript abundance itself

To test whether *ELF3* expression is present and varies between HIF sister lines, as well as to further understand the phenotypic differences between Barke *ELF3*_*Hv*_ and wild *ELF3*_*Hsp*_ allele-carrying HIF sister lines originating from the same wild donor, we analysed diurnal expression of *ELF3* itself as well as its downstream genes *HvCO1*, *HvGI*, *PPD-H1*, *VRN-H3*/*HvFT1*, and *VRN-H1*. Plants were grown under 16 h/8 h light/dark cycles with day/night temperatures of 20 °C/18 °C, and leaf samples were collected every 4 h over a 24 h period on day 17. The two HIF pairs 10_003 and 10_190 were selected due to the strong effect differences between HIF sister lines in 10_190 and the non-existent effects in 10_003, for which only assumptions could be made until now. Bowman and the *elf3* mutant BW290 were chosen for comparison reasons.

**Fig. 5.**
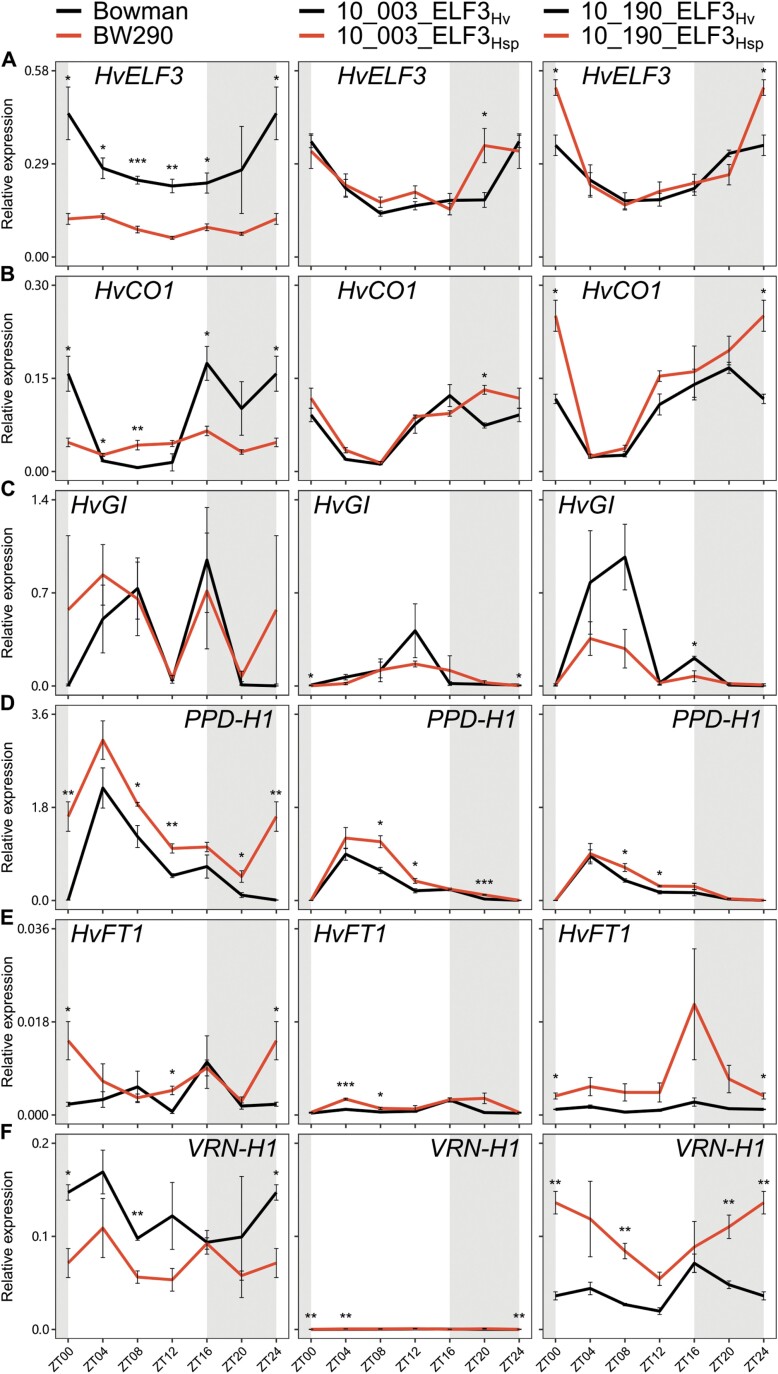
Transcript levels of flowering time genes in Bowman, BW290, and HIF pairs 10_003 and 10_190. Diurnal gene expression of *HvELF3* (A), *HvCO1* (B), *HvGI (C), PPD-H1* (D), *HvFT1* (E), and *VRN-H1* (F) was measured every 4 h in plants grown under standard conditions (LDs with 20/18 °C day/night temperatures). Grey-shaded areas indicate darkness. Expression levels were normalized to *HvGAPDH* and *PTF*. Error bars indicate the SE of the mean (*n*=3) of three biological replicates. Asterisks above lines indicate significant differences (two-sided Student’s *t*-test, *P<0.05, **P<0.01, and ***P<0.001).

Bowman displayed generally higher *HvELF3* transcript abundance compared with the mutant BW290 in diurnal conditions except ZT20 (Fig. 5A), similar to previous observations under short days (Faure *et al.*, 2012). However, this contradicts another previous report using a different *elf3* mutant BW289 under 12 h/12 h light/dark cycles (Zakhrabekova *et al.*, 2012). As both BW289 and BW290 lack functional HvELF3 proteins (Zakhrabekova *et al.*, 2012), the discrepancy in *HvELF3* transcript abundance is not expected to influence downstream signalling pathways. In contrast, the transcript abundance of *HvELF3* was not different between *ELF3*_*Hv*_ and *ELF3*_*Hsp*_ in HIFs 10_003 and 10_190, except for occasionally detected increased *HvELF3* expression in *ELF3*_*Hsp*_ lines (10_003 at ZT20 and 10_190 at ZT00/24) (Fig. 5A). These data suggest that the observed phenotypic variation between *ELF3*_*Hv*_ and *ELF3*_*Hsp*_ in HIF 10_190 was not or at least not mainly due to transcript abundance of *HvELF3,* which is supported by the observation that the promotors of *ELF3*_*Hv*_ and *ELF3*_*Hsp*_ are identical (Supplementary Table S12).

Interestingly, the rhythmic expression pattern of *HvCO1* in Bowman was not detected in BW290 (Fig. 5B). The transcript abundance of *HvCO1* in BW290 was generally higher during the light period, but lower during the dark period than in Bowman. A similar expression profile of *CO1* was found in a study with an *elf3* mutant in *B. distachyon* (Bouché *et al.*, 2021), a grass closely related to barley, for which the conserved role of *ELF3* could be shown (Huang *et al.*, 2017; Bouché *et al.*, 2021). In that study, *CO1* expression seemed to be down-regulated in the mutant during the night as in BW290. However, this strong effect of the *elf3* mutation was not observed in the HIF pairs. Both sister lines in HIFs 10_003 and 10_190 displayed rhythmic *HvCO1* expression similar to Bowman, with *ELF3*_*Hsp*_ lines showing slightly higher expression at ZT20 in 10_003 and at ZT00/24 in 10_190. In Arabidopsis, *elf3* mutations induce *CO* expression by stabilizing GI (Yu *et al.*, 2008); meanwhile the transcription of *GI* is directly repressed by the EC (Ezer *et al.*, 2017). Although such ELF3–GI–CO connections are not validated in barley, *HvGI* expression was predicted to be repressed by the EC component HvLUX1 (Müller *et al.*, 2020). However, almost no difference was observed in *HvGI* transcript abundance between Bowman and BW290 or between HIF sister lines (Fig. 5C). The observation in BW290 again contradicts the induced *HvGI* expression in a different *elf3* mutant, BW289 (Zakhrabekova *et al.*, 2012). Nevertheless, we could not rule out the potential effect of *ELF3* on HvGI stability, which might in turn affect *HvCO1* expression.

Consistent with previous reports (Faure *et al.*, 2012; Ejaz and von Korff, 2017), the transcript abundance of *PPD-H1* increased in BW290 compared with Bowman at various time points. Such induction of *PPD-H1* expression was also observed in ELF3_Hsp_ compared with ELF3_Hv_, except for ZT00/24 in both HIFs and ZT20 in 10_190 (Fig. 5D). Although 10_003 and 10_190 carry differently fixed homozygous *PPD-H1*_*Hv*_/*PPD-H1*_*Hsp*_ alleles (Supplementary Table 17) responsible for later/earlier flowering, respectively (Maurer *et al.*, 2016), this does not seem to affect *PPD-H1* transcript abundance itself; as reported in an earlier study (Turner *et al.*, 2005). Our data suggest that ELF3_Hsp_ in HIFs 10_003 and 10_190 induces *PPD-H1* expression regardless of its allelic differences between HIF pairs.

As expected, the increased transcript abundance of *PPD-H1* was associated with the transcriptional induction of its putative target *HvFT1* in BW290 (as in Ejaz and von Korff, 2017), 10_003_ELF3_Hsp_, and 10_190_ELF3_Hsp_ (Fig. 5E). Although *HvFT1* expression is mainly controlled by *PPD-H1* under LDs, other players such as *HvCO1* and/or *VRN-H1*, which both up-regulate *HvFT1*, might be involved (Turner *et al.*, 2005; Hemming *et al.*, 2008; Campoli *et al.*, 2012a). The up-regulation of *HvFT1* is associated with the expression of barley floral meristem identity genes *VRN-H1*, *BARLEY MADS-box 3* (*HvBM3*), and *HvBM8*, initiating inflorescence development (Digel *et al.*, 2015; Ejaz and von Korff, 2017; Gol *et al.*, 2021). Indeed, transcript abundance of *VRN-H1* was induced at various time points in 10_190_ELF3_Hsp_ compared with ELF3_Hv_ (Fig. 5F), consistent with its early heading phenotypes under both field and greenhouse conditions (Figs 2, 3). However, the *VRN-H1* transcript abundance was slightly reduced in BW290 compared with Bowman at ZT00/24 and ZT08. We would expect that the altered transcript abundance of *PPD-H1* and *HvFT1* in BW290 would influence the expression of other floral regulators, such as *HvBM3* and *HvBM8*, which were more prominently correlated with *HvFT1* (Ejaz and von Korff, 2017; Gol *et al.*, 2021). Although transcript abundance of *VRN-H1* was induced in 10_003_ELF3_Hsp_ compared with ELF3_Hv_, HIF 10_003 displayed overall low *VRN-H1* expression compared with Bowman, BW290, and HIF 10_190. As this HIF carries a homozygous *VRN-H1*_*Hsp*_ from the assumed winter type wild barley donor (Supplementary Table S17), the expression of *VRN-H1* was probably repressed due to the lack of vernalization. Therefore, these data can explain that the observed phenotypic differences between HIFs 10_003 and 10_190 in the field (Fig. 2) are due to different *VRN-H1* alleles.

Overall, these results confirm *ELF3* expression in the HIF lines and, as shown in previous reports with *elf3* mutants, also show differences in the expression of barley flowering time genes between HIF sister lines; although this is unlikely to be due to *ELF3* transcript abundance itself. As expected, effects of the loss-of-function *elf3* mutant on gene expression were stronger than between the natural *ELF3* variants in the HIF pairs, consistent with the respective phenotypes in the greenhouse experiment (Fig. 3). Furthermore, these results confirm the earlier assumption that the effects of *ELF3* depend on the genetic background, consistent with its upstream role in barley flowering time regulation (Faure *et al.*, 2012; Boden *et al.*, 2014). Therefore, comparing gene expression in the present HIF pairs is rather complicated since the HIFs originating from the same family are diverse in the remaining genome. Although it is very likely that *VRN-H1* is the reason for the huge differences between HIFs in family 10, further different homozygous alleles between HIFs 10_003 and 10_190 for *PPD-H1*, *CEN*, *QFt.HEB25-4a*, *VRN-H3/FT1* (Supplementary Table S17), and two *GI*-related genes, *LUX*, *ELF4*, and *PIF4* (Supplementary Table S19), several of which have been shown to interact with or be affected by ELF3, make a comprehensive comparison between these two HIFs hardly possible. This could be facilitated by the development of double HIFs (e.g. segregating at *ELF3* and *PPD-H1* or *PIF4*), with an isogenic background, in the future. Here, the concept is to detect a HEB line which is heterozygous at both loci of interest and select segregating offspring genotypes in all four possible combinations of wild and elite alleles at these two loci (i.e. *PPD-H1*_*Hv*_/*ELF3*_*Hv*_, *PPD-H1*_*Hv*_/*ELF3*_*Hsp*_, *PPD-H1*_*Hsp*_/*ELF3*_*Hv*_, and *PPD-H1*_*Hsp*_/*ELF3*_*Hsp*_). Future experiments should then include phenotyping and diurnal gene expression analysis to examine whether the different combinations of wild and cultivated alleles of the respective genes show distinct effects on flowering time or other traits as well as changes in diurnal expression of themselves and downstream genes, making further statements about their interactions in a more isogenic background possible.

### W669G substitution probably affects protein structure of ELF3 and might induce disorder-driven phase separation events forming local nano-compartments

Since gene expression data suggested that the observed phenotypic variation between sister lines in HIF 10_190 was not due to *HvELF3* transcript abundance itself (Fig. 5A), it may be due to differences on the protein level, so we evaluated a potential impact of the minimal amino acid change W669G in the protein sequence between ELF3_Hv_ of Barke (ELF3_Barke_) and ELF3_Hsp_ of HID_102 (ELF3_HID102_), the exotic donor of HEB family 10. Since HIF sister lines of 10_190 are nearly isogenic (<1% of segregation, Supplementary Table S18), and it is the only HIF harbouring only one single amino acid difference between wild ELF3 and Barke ELF3 (Fig. 4C), which is simultaneously showing strong phenotypic differences between HIF sister lines (Fig. 2), it is a good basis to further investigate the influence of W669G regarding its effects on the ELF3 protein structure and thereby possible influences on the phenotype. Of course, it would also be interesting to investigate the influence of amino acid substitutions other than W669G, especially when there are only differences for individual amino acids between two ELF3 proteins. One example is ELF3 in family 3 (HID_055), because it only varies from the cultivated ELF3 of Bowman at position 709 (G709W). In the context of the HIF comparison, however, the association of ELF3 variants with flowering time and crop performance would be hampered as we do not have Bowman *ELF3* alleles for comparison present in HIFs. However, this could be a future approach to create HIFs based on crossings of HID_055 and Bowman to investigate the impact of G709W on the phenotype.

**Fig. 6.**
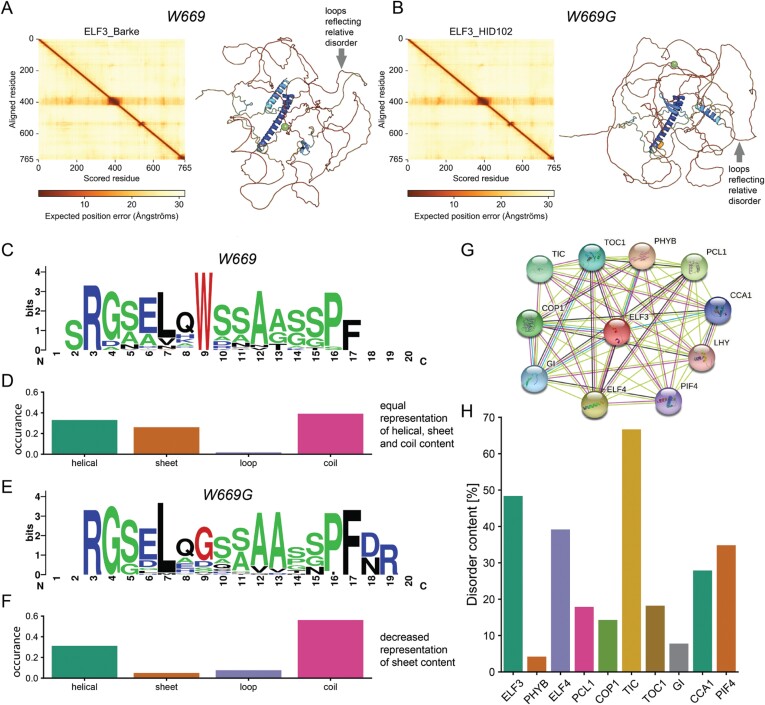
Sequence and structural analysis of the W669G substitution. (A and B) Alphafold2 prediction of the Barke and HID_102 sequence of ELF3. Models are coloured by their respective plDDT scoring, which indicates the reliability of the derived model. The Cα atom of the mutation site is highlighted as a green sphere. Left plots in (A) and (B) are standard analysis plots of Alphafold-derived models reflecting a per-residue estimate of its confidence on a scale from 0 to 100 and translated into positional ambiguity in 3D space (from 0 to 30 Å as shown in the scale bar below each plot) as described in Jumper *et al.* (2021). This measure is translated into plDDT scores and correlates with intrinsic disorder (Jumper *et al.*, 2021). Red regions in the depicted Alphafold models (loops) show probable regions of high disorder content. (C–F) Local analysis of homologous structures. (C, E) Weblogo of the identified homologues for structures for Barke (W669) and substituted HID_102 (W669G) sequence. The site of the amino acid substitution at position 9 is highlighted in red, residues prone to be in disordered regions are highlighted in green, and charged residues in blue. (D, F) Statistical occurrence of secondary structure element in the identified structures. (G and H) Interaction analysis and disorder prediction for ELF3 and interaction partners. (G) STRING network for ELF3 from Arabidopsis. (H) Disorder content of the barley proteins interacting with ELF3.

In order to evaluate the potential impact of W669G on the protein level between ELF3_Barke_ and ELF3_HID102_, we performed a sequence/structure-based analysis to identify possible effects of the W669G substitution observed in HvELF3 at the protein level. Note here that computational and modelling results provide a valid and experimentally testable hypothesis and do not comprise proof. Based on InterPro (Blum *et al.*, 2021), no domain is known for barley ELF3, or for the better annotated Arabidopsis homologue. Sensitive Markov search with HHPred (Gabler *et al.*, 2020) revealed helical content with low confidence for residues 373–395. Utilizing the state-of-the-art Alphafold2 algorithm (Jumper *et al.*, 2021), the entire structure of ELF3_Barke_ and ELF3_HID102_ with the substitution was predicted (Fig. 6A, B). Interestingly, high disorder content is predicted, and, as expected, the W669G substitution is also localized in those regions (Fig. 6A, B).

We next asked if the local structural preferences of this substitution could be altered. To answer this, we selected the subsequence 661–680, and structural homologues were identified by BLASTp. Identified results were filtered, using a threshold of a minimum of five resolved residues in the determined structure, and annotated secondary structure was retrieved. In total, 52 and 34 structures were identified for the Barke and HID_102 sequence regions, respectively (Supplementary Table S21). Notably, the identified structural homologues for the Barke sequence have a conserved Trp at position 9, whereas HID_102 showed lower conservation of the substituted Gly (Fig. 6C–F). Analysing the secondary structure content of identified homologues revealed deviations in local folding preference (Fig. 6D, F; Supplementary Table S21). For Barke, the region folds in α-helical, β-sheet, and coil conformations with equal occurrence. For the variant W669G in HID_102, a dramatic decrease in β-sheet occurrence is derived (Fig. 6F). Gly, besides Pro, is a known β-sheet breaker (Minor and Kim, 1994; Smith *et al.*, 1994), corroborating this observation with possible effects on higher order hydrogen-bonding patterns.

ELF3 contains a high content of unstructured/disordered protein regions (Fig. 6A), and locally these regions can transition equally to various secondary structure elements as shown by our analysis (Fig. 6D, F). Disordered protein regions are often linked to phase separation events (Majumdar *et al.*, 2019), forming local nano-compartments in the cell. Given that AtELF3 itself phase-separates (Jung *et al.*, 2020), this substitution could well affect the phase separation behaviour of ELF3 in barley (i.e. the switch between the active and the inactive state), as shown for Arabidopsis in the context of temperature sensitivity (Jung *et al.*, 2020). The causal relationship and functional consequences of phase separation *in vivo* are very elusive (McSwiggen *et al.*, 2019), so there are still many open questions regarding potential effects and functional consequences of ELF3 phase separation and nano-compartment formation in barley. Another, less obvious effect of W669G could be in the context of its local cellular interactions, and we therefore analysed its annotated cellular community. Based on the STRING (Szklarczyk *et al.*, 2021) entry of the homologous protein from Arabidopsis, AtELF3 has 10 described interaction partners identified using the default criteria (Fig. 6G). We identified the respective barley proteins using BLASTp and analysed the disorder content utilizing the MobiDB-lite algorithm (Necci *et al.*, 2017) (Supplementary Table S22). The majority of the interaction partners also show high disorder content, with a mean value of 28% when considering their full sequences (Fig. 6H). Based on this, the effect of this W669G substitution might not only affect the phase separation behaviour of ELF3, but might also be involved in disorder-driven phase separation events within its cellular community.

The substitution of residue 669 from Trp to Gly might play an essential role in regulating a function in a higher order assembly. This is because (i) the Trp-containing sequence can adopt all types of secondary structure, whereas the Gly substitution induces a reduced β-sheet content due to the sheet-breaking properties of Gly. This might directly affect secondary structure transitions, needed in disordered regions to adapt for self-interacting (as in the case of AtELF3; Jung *et al.*, 2020) or/and interacting with different interaction partners and thereby influencing complex composition and higher order community 3D architecture (Kim and Han, 2018). (ii) Trp contains a delocalized π-electron system in its side chain, and is thereby able to form π–π, π-stacking, and cation–π interaction networks. This interaction seems to play an essential role in the process of phase separation (Vernon *et al.*, 2018). The analysed substitution might thereby directly affect the properties underlying nano-compartment formation and, ultimately, regulate a functional complex to perform functions with distinct phenotypic consequences.

However, given that *Hsp* alleles of all other HIFs also share this substitution (Fig. 4) and that 10_003 harbours exactly the same *ELF3*_*Hsp*_ allele without showing the same strong phenotypic effects underline the dependency of the *ELF3* effect on the genetic background, shaping a complex interaction network.

## Conclusions and future prospects

In this study, we validated QTL effects from previous barley field studies that were attributed to a genomic region that included *ELF3* (Maurer *et al*., 2015, 2016; Herzig *et al.*, 2018). We determined novel exotic *ELF3* alleles and made use of nearly isogenic barley HIF pairs that segregated for the *ELF3* gene. The HIF pairs confirmed variation between *ELF3* alleles and genotype×environment interaction across the studied years. A greenhouse experiment confirmed the major results of the field trials. HIF pair 10_190 was especially promising, showing strong effects without yield losses in the field experiment and even stronger effects in the greenhouse. For natural *ELF3* variants, we found variation regarding flowering time gene expression in barley. However, the phenotypic trait differences could not be explained by differences in *ELF3* transcript abundance itself, but may be explained by the substitution of a single amino acid, which was predicted to influence ELF3 protein structure, thereby possibly affecting the properties underlying nano-compartment formation of ELF3 and potentially also affecting its interaction partners inside the cell. This emphasizes the influence of further factors impacting the phenotypic *ELF3*_*Hsp*_ allele effects, such as further variation at the protein level or in the remaining genome, for example alleles at other genes like *PPD-H1*, *PIF4*, or *VRN-H1*, for some of which a modified expression could already be shown (Fig. 5). Due to the central role of ELF3 in the circadian clock with manifold protein interactions, in future experiments additional HIFs differing in their genomic background should be selected and characterized to shed further light on the control of plant development by interacting substitutions at critical amino acid positions of the ELF3 protein. Ultimately, this study confirmed that HIFs can be a useful tool to characterize and validate allelic effects from previous QTL studies. We have shown that the selection of HIFs with a fixed genomic background is crucial to obtain significant results. Furthermore, we propose double HIFs, simultaneously segregating at two loci, as a valuable option to investigate epistatic effects or dependencies between interacting genes. The identification of promising *ELF3* alleles for improvement of developmental and yield-related traits in barley is important for barley breeding, especially for adaptation of elite barley to climate change-related stresses.

## Supplementary data

The following supplementary data are available at *JXB* online.

Table S1. IBD genotype data from the Infinium iSelect 50k SNP chip of pre-selected BC_1_S_3:8_ lines with a heterozygous *ELF3* locus.

Table S2. Markers used for selection of HIF sister lines.

Table S3. IBD genotype data from the Infinium iSelect 50k SNP chip of all HIFs used in the field trial (with a homozygous *ELF3* locus).

Table S4. Day lengths and weather conditions during the respective growth periods in 2019 and 2020.

Table S5. Repeatabilies (Rep) and heritabilites (H^2^).

Table S6. Primers used for PCR and sequencing of *ELF3* coding and promotor sequence and primers used for gene expression analysis.

Table S7. Raw data and BLUEs of all investigated traits with significant differences between HIF sister lines.

Table S8. Descriptive statistics of all investigated traits based on BLUEs for both years separately.

Table S9. ANOVA results of phenotypic data for genotype, year, and genotype×year interactions.

Table S10. Descriptive statistics for each HIF sister line per year.

Table S11. ANOVA results of phenotypic data for the image-based greenhouse experiment.

Table S12. Coding sequences of all 25 wild donors of HEB-25, Barke, Bowman, and BW290 and the promotor sequence of *ELF3* in HIF sister lines of 10_190.

Table S13. Protein sequences of all 25 wild donors of HEB-25, Barke, Bowman, BW290, and Morex.

Table S14. Variation in amino acids of all 25 wild donors of HEB-25, Barke, Bowman, BW290, and Morex.

Table S15. Variation in coding sequences of all 25 wild donors of HEB-25, Barke, Bowman, and BW290, and comparison with variation already described in the literature.

Table S16. Summarizing table of all ELF3 protein sequence polymorphisms present in the field trials and their respective phenotypic effects in both years.

Table S17. IBD genotype data from the Infinium iSelect 50k SNP chip of all HIFs for the eight major flowering time loci.

Table S18. Segregation of HIF sister lines in base pairs and as a percentage of the whole barley genome of 5.1 Gbp including the ELF3 region.

Table S19. IBD genotype data from the Infinium iSelect 50k SNP chip of a selection of genes that are also important for flowering time control in barley.

Table S20. Genes in segregating regions of line 10_190 extracted from Barlex (IPK Gatersleben).

Table S21. Data from the local sequence analysis, including the identified pdb file, chain, residue range, and occurrence of secondary structure elements.

Table S22. Accession codes for the barley homologues and the disorder content prediction.

Fig. S1. Weather data.

Fig. S2. Boxplots for all traits and both years separately.

Fig. S3. Correlations of traits for 2019 and 2020 separately.

Figure S4. Trait differences between the two sister lines of each HIF pair (*ELF3*_*Hsp*_ compared with *ELF3*_*Hv*_) across years.

Fig. S5. Segregating regions between HIF sister lines.

Protocol S1. Repeatabilities and heritabilities. Correlations. Impacts of the environment on phenotypes.

erad127_suppl_Supplementary_Tables

erad127_suppl_Supplementary_Material

## Data Availability

All relevant data are included in the supplementary files. Protein sequence/structure analysis scripts can be made available upon request.

## Abbreviations

BC_1_S_3_: backcross one, selfing three generation
*BM3/8*: *BARLEY MADS-box3/8*
*CO*: *CONSTANS*
EAR: ears m^–2^
EC: evening complex
*ELF3*: *EARLY FLOWERING 3*
*FT*: *FLOWERING LOCUS T*
*GI*: *GIGANTEA*
GNE: grain number per ear
HEA: time to heading
HEI: plant height
*LUX*: *LUX ARRHYTHMO*
MAT: time to maturity
NAM: nested association mapping
*PIF4*: *PHYTOCHROME-INTERACTING FACTOR 4*
RIP: ripening phase
SEL: shoot elongation phase
SHO: shooting
SNP: single nucleotide polymorphism
TGW: thousand grain weight
YLD: grain yield.
